# Correction: Flavin-adenine-dinucleotide gold complex nanoparticles: chemical modeling design, physico-chemical assessment and perspectives in nanomedicine

**DOI:** 10.1039/d3na90084c

**Published:** 2023-08-14

**Authors:** Celia Arib, Nadia Bouchemal, Maria Barile, Didier Paleni, Nadia Djaker, Nathalie Dupont, Jolanda Spadavecchia

**Affiliations:** a CNRS, UMR 7244, CSPBAT, Laboratoire de Chimie, Structures et Propriétés de Biomatériaux et d'Agents Thérapeutiques Université Paris 13, Sorbonne Paris Nord, 1 Rue Chablis 93000 Bobigny France jolanda.spadavecchia@univ-paris13.fr; b Department of Biosciences, Biotechnology and Biopharmaceutics, University of Bari “Aldo Moro” Via Orabona 470126 Bari Italy; c BioEVEN start-up 75 Rue de Lourmel 75015 Paris France

## Abstract

Correction for ‘Flavin-adenine-dinucleotide gold complex nanoparticles: chemical modeling design, physico-chemical assessment and perspectives in nanomedicine’ by Celia Arib *et al.*, *Nanoscale Adv.*, 2021, **3**, 6144–6156, https://doi.org/10.1039/D1NA00444A.

The authors regret that a panel in Fig. S3 was incorrect, depicting FAD release in PEG-AuNPs at pH 4 and pH 7 (panels B and B1 accidentally showed the same data rather than the data at different pH values). The original data has been provided and the corrected panels B (FAD release at pH 7) and B1 (FAD release at pH 4) are shown below.


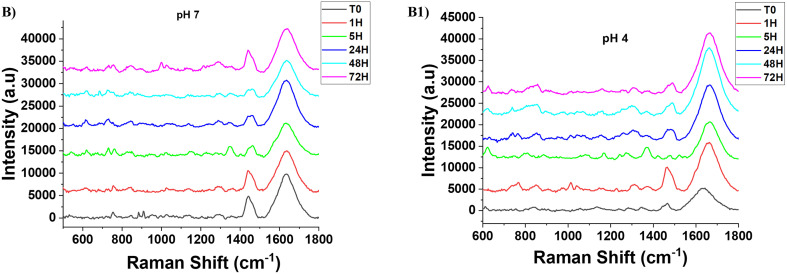
 **Fig. S3** Monitoring release of FAD in PEG-AuNPs at (B) pH 7 and (B1) pH 4 by Raman spectroscopy.

An independent expert has viewed the corrected images and has concluded that they are consistent with the discussion and conclusions presented.

The Royal Society of Chemistry apologises for these errors and any consequent inconvenience to authors and readers.

## Supplementary Material

